# Serum neurofilament light protein as a biomarker in Niemann-Pick disease, type C1

**DOI:** 10.1016/j.gimo.2025.103443

**Published:** 2025-07-07

**Authors:** Niamh X. Cawley, Ruyu Zhou, Avani Mylvara, Cameron J. Padilla, Derek Alexander, Nicole Farhat, Carolina Alvarez, Antony Cougnoux, Elizabeth Berry-Kravis, Stephanie M. Cologna, Fang Liu, Forbes D. Porter

**Affiliations:** 1 Section on Molecular Dysmorphology, Division of Translational Medicine, *Eunice Kennedy Shriver* National Institute of Child Health and Human Development, National Institutes of Health, Bethesda, MD; 2 Applied and Computational Mathematics and Statistics, University of Notre Dame, South Bend, IN; 3 Departments of Pediatrics, Neurological Sciences, and Anatomy and Cell Biology, Rush University Medical Center, Chicago, IL; 4 Department of Chemistry and Laboratory of Integrative Neuroscience, University of Illinois Chicago, Chicago, IL

**Keywords:** Hydroxypropyl-β-cyclodextrin, Miglustat, Neurofilament light, Niemann-Pick disease, Serum biomarker

## Abstract

**Purpose:**

Niemann-Pick disease, type C1 (NPC1) is a fatal, neurodegenerative disease caused by pathological variants in *NPC1*. Analysis of serum neurofilament light (NfL), a marker of neuronal damage, could be useful as a biomarker for patient monitoring and clinical trial design.

**Methods:**

We measured NfL levels in serum samples from 118 well-characterized individuals with NPC1 and analyzed them with respect to clinical measures and treatment status.

**Results:**

The results show a 6.1-fold increase in serum NfL in individuals with NPC1 compared with age-appropriate controls. Moreover, serum NfL levels showed a significant positive correlation with age of neurological symptom onset and the annual severity increment score. Serum NfL levels were also positively correlated with the 17- and 5-domain NPC Neurological Severity Scores. Longitudinal analyses reveal a 26% reduction in serum NfL levels in individuals on miglustat, a therapeutic drug used off-label for the treatment of NPC1 in the United States of America. Effectiveness of intrathecal hydroxypropyl-β-cyclodextrin treatment may be more beneficial in younger individuals. To help inform clinical trial design, our modeling predicts that a measurable reduction of serum NfL levels might be observed after 8 months of treatment with a potential drug exhibiting 10% to 20% efficacy.

**Conclusion:**

Our data suggest that NfL may be a useful serum biomarker for NPC1.

## Introduction

Biomarker research is an important component of the study of human disease. With the identification of biomarkers, clinicians and affected individuals can make informed decisions about therapeutic interventions and counseling. This is particularly important for diseases of the central nervous system (CNS), for which identification of biomarkers of neuroinflammation or neurodegeneration are crucial for following both disease progression and clinical response.

Niemann-Pick disease, type C1 (NPC1, OMIM 257220) is an ultrarare, fatal, lysosome storage disorder that affects the peripheral and central nervous systems. In the childhood form it often presents with hepatosplenomegaly and liver dysfunction, but as the disease progresses, cerebellar ataxia, supranuclear vertical gaze palsy, and cognitive impairment develop, generally leading to death in the second decade of life.^1^^,^^2^ NPC1 is an autosomal recessive disorder with an incidence rate for the juvenile form estimated to be at ∼1/100,000.^1^^,^^3^ A similar disorder, NPC2, (OMIM 607625) is seen with defective NPC2; however, the rate of incidence is estimated at ∼1/2,000,000.^3^

NPC1 is a transmembrane protein that functions to transport cholesterol out of the endolysosomal lumen. Pathogenic variants of *NPC1* result in the impaired function of NPC1, leading to accumulation of unesterified cholesterol within the lysosomal/endosomal compartments and reduced cellular cholesterol bioavailability. This results in cellular stress and cell death, particularly for neurons. Purkinje neuron loss in the cerebellum is the primary cause of cerebellar ataxia, a prominent neurological symptom of NPC1 disease.^1^

The age of NPC1 neurological disease onset and disease severity is highly variable, ranging from infancy to adulthood. Combined with its rarity and relatively slow progression,^1^ it is difficult to develop effective therapies for regulatory approval. Miglustat, an inhibitor of glucosylceramide synthase, is approved for the treatment of NPC1 by the European Medicines Agency, but it has not been labeled for stand-alone treatment of NPC1 by the Food and Drug Administration (FDA) despite substantial real-life data in support.^4^, ^5^, ^6^ Recently, the FDA approved the use of arimoclomol (Myplyffa, Zevra Therapeutics) in combination with miglustat as the first approved treatment for NPC1 in the United States.^7^ N-acetyl-L-leucine (AQNEURSA, IntraBio Inc) was also recently approved by the FDA as the first stand-alone treatment for NPC1.^8^ Studies using intrathecal (IT) hydroxypropyl-beta-cyclodextrin (HPβCD, also known as adrabetadex) as a treatment modality for NPC1 have shown promise in clinical trials (NCT01747135, NCT02534844) and are ongoing.

In the cerebrospinal fluid (CSF), proteins that are altered in individuals with NPC1 have been identified by targeted and proteomic screens. Proteins with altered expression include glutathione S-transferase alpha, superoxide dismutase, and FABP3,^9^ amyloid-β and TAU,^10^ calbindin D and FABP3,^11^ UCHL-1,^12^ NPY and Cathepsin D,^13^ NfL^14^, ^15^, ^16^ and MAPT, CHIT1, CALB1 PARK7, CALB2/calretinin, CHI3L1/YKL-40, MIF, CCL18, and ENO2.^16^ Several of these proteins, eg, NfL, UCHL-1, CHI3L1, MIF, CALB2, NPY, and ENO2, showed significant correlations with clinical disease parameters or therapeutic responses. Another molecule, 24-(S) hydroxycholesterol (24(S)-HC), a specific metabolite of cholesterol in neurons, is a proximal pharmacodynamic marker of target engagement.^17^ Measurement of 24(S)-HC in plasma and CSF was used to track disease treatment efficacy with IT HPβCD in a phase 1/2 clinical trial.^11^ However, although the results from plasma are useful, they were variable. In contrast, the changes in the levels of 24(S)-HC in CSF were more dynamic.

In the blood, several molecules exist that are considered blood-based diagnostic biomarkers of NPC1 such as 3b,5a,6b-cholestane-triol, N-(3β,5α,6β-trihydroxy-cholan-24-oyl)-glycine and N-palmitoyl-O-phosphocholineserine.^18^ Indeed, N-(3β,5α,6β-trihydroxy-cholan-24-oyl)-glycine, a bile acid metabolite, has shown promise for newborn screening.^19^ However, production of these metabolites by peripheral organs does not reflect necessarily neurological disease burden or treatment in the brain. Identifying a blood-based biomarker that reflects neurological status in NPC1 would greatly improve disease monitoring and help in therapeutic trial design.

Previous work has shown increased neurofilament light (NfL) protein in biofluids, such as CSF^15^^,^^20^ and plasma,^14^^,^^21^^,^^22^ in individuals with NPC1, providing a strong foundation for investigating NfL serum levels in a larger cohort of clinically well-characterized individuals with NPC1 both at baseline and over time. NfL is a protein component of microtubules found as part of the cytoskeleton of neurons. In neurodegenerative diseases, such as Alzheimer disease, Parkinson disease, multiple sclerosis, and amyotrophic lateral sclerosis (ALS), NfL has been measured as a readout of neuronal damage.^23^, ^24^, ^25^, ^26^, ^27^, ^28^ Indeed, it is routinely used to measure and track recovery from traumatic brain injury.^29^ Recently, the FDA approved antisense oligonucleotide treatment for superoxide dismutase 1(SOD1)-dependent ALS.^30^^,^^31^ The approval was based on reduced plasma NfL levels associated with treatment, laying the framework for the possible use of NfL as a readout measure for clinical trials of NPC1 and other neurodegenerative diseases.

In this study, we applied Simoa based nanotechnology to quantify levels of NfL in archived cross-sectional and longitudinal serum samples from a large cohort of individuals with NPC1. We investigated whether serum levels of NfL correlated with clinical aspects of NPC1 and if these biomarkers responded to therapeutic interventions.

## Materials and Methods

### Serum collection, clinical phenotyping, and participants

Clinical data and biomaterials were obtained from individuals with NPC1 enrolled in a natural history/observational study (NCT00344331), a phase 1/2 and a phase 2/3 trial of intrathecal hydroxypropyl-beta-cyclodextrin (IT HPβCD) (NCT01747135, NCT02534844, respectively), conducted at the National Institutes of Health Clinical Center, Bethesda, Maryland, USA. NPC Neurological Severity Score (NSS) is utilized to measure disease burden at each visit, with a 17-domain disease severity rating (9 major and 8 minor domains).^32^ A 5-domain scoring protocol^33^ was also used and is highly correlated with the 17-domain score; the 5 domains (ambulation, fine motor, speech, swallow, and cognition) are considered by clinicians and guardians to be of clinical importance.^33^ The NPC NSS allows for assessment of disease burden and progression. An annual severity increment score (ASIS) was calculated by normalizing the 17-domain NPC NSS to age in years.^34^ Age of neurological onset (ANO) was determined by systematic review of the clinical history. ASIS and ANO are measures of disease severity.

Sera were collected at each visit in a 10mL BD Vacutainer (Red Top) Serum Tube, following National Institutes of Health Clinical Center guidelines. Whole blood samples were kept on ice, no longer than 45 minutes after collection. Whole blood was centrifuged at 1500 × g for 10 minutes at 4 °C. Serum was immediately extracted, aliquoted, and stored at −80 °C.

The serum NfL data set contains records of 155 samples from 118 individuals having available serum NfL data from at least one visit. Sera from 24 age-appropriate pediatric individuals were used as the control group (Table 1).

**Table 1:** Summary statistics of baseline variables

Variable	Control (*n* = 24)	NPC1 (*n* = 118)	Miglustat (*n* = 55)	No Miglustat (*n* = 63)
Age (years)				
Mean ± SD	11.1 ± 4.8	15.0 ± 14.8	14.1 ± 11.8	15.7 ± 17.1
Median (IQR)	11.0 (7.4-15.1)	10.6 (3.8-20.0)	11.8 (6.1-17.6)	10.5 (1.9-21.2)
Range	2.1 - 19.4	0.3-68.1	0.8-62.5	0.3-68.1
Sex (frequency (%))				
Male	16 (66.6%)	56 (47.5%)	26 (47.3%)	30 (47.6%)
Female	8 (33.3%)	62 (52.5%)	29 (52.7%)	33 (52.4%)
17-domain severity score	NA			
Mean ± SD		14.2 ± 10.7	14.0 ± 10.0	14.3 ± 11.3
Median (IQR)		14.0 (5-20)	13.0 (7.5-20.0)	14.0 (3.0-22.5)
Range		0-46	0-44	0-46
5-domain severity score	NA			
Mean ± SD	NA	7.5 ± 6.4	6.8 ± 5.5	8.0 ± 7.0
Median (IQR)		7.0 (2-12)	6.0 (3.0-8.5)	7.0 (1.0-13.0)
Range		0-25	0-22	0-25
ANO (years)^a^	NA			
Mean ± SD		8.3 ± 10.6	7.5 ± 8.9	9.1 ± 12.0
Median (IQR)		5.0 (2-9)	5.3 (2-9)	5 (2-9)
Range		0.5-52	0.5-52	0.5-51
ASIS^b^	NA			
Mean ± SD		1.8 ± 2.3	1.6 ± 1.8	2.1 ± 2.7
Median (IQR)		1.1 (0.6-2.1)	1.0 (0.5-1.9)	1.2 (0.6-2.1)
Range		0.06-14.7	0.06-6.9	0.15-14.7
Treatment (frequency (%))	NA	55 (46.6%)		
Miglustat		8 (6.8%)	5 (9.1%)	3 (4.8%)
IT HPβCD arimoclomol		3 (2.5%)	3 (5.5%)	0 (0%)

*ANO*, age of neurological onset; *ASIS*, annual severity increment score; *HPβCD*, 2-hydroxypropyl-β-cyclodextrin (VTS270, adrabetadex); *IT*, intrathecal; *N/A*, not applicable; *NSS*, neurological severity score.

a *n* = 109 for the NPC1 cohort, *n* = 52 for miglustat at baseline, and *n* = 57 for no miglustat at baseline. Nine individuals had not yet developed neurological symptoms.

b *n* = 104 for the NPC1 cohort, *n* = 52 for miglustat at baseline, and *n* = 52 for no miglustat at baseline. ASIS could not be determined for 14 individuals who had 17-domain scores of 0.

### Quantification of NfL levels in serum

Serum NfL levels were measured by the Neuro4-Plex enzyme-linked immunoassay using Simoa technology on the Quanterix SR-X platform in a 96-well plate format (Quanterix). The Neuroplex 4 assays also provided data on serum GFAP, TAU and UCHL1 in addition to NfL levels. Serum was thawed on ice, diluted 1:4 with sample diluent and assayed in duplicate. The plates were processed using the 2-step digital immunoassay as outlined in the manufacturer’s protocol. All plates had 2 internal controls: low (C1) and high (C2) NfL ranges. The internal controls, from 11 assays over 2 years had inter-assay coefficient of variation (%) of 17.0% for NfL C1 and 13.4% for NfL C2. In a separate set of assays, CSF NfL levels were also measured using the Neuro4-Plex enzyme-linked immunoassay Simoa technology on the Quanterix SR-X platform (Supplemental Data).

### Statistical analysis methods

Variables collected at the first visit with available serum NfL data were considered as the baseline for each patient and were summarized by various descriptive statistics as relevant for the variable, such as frequencies and percentages for categorical variables or mean ± SD, median and interquartile range, and range for numerical variables. The distribution of serum NfL (pg/mL) was assessed for normality and all analyses were performed with log-transformed data. Spearman correlation coefficients are reported with 95% CIs and *P* values, between serum log_10_NfL levels at baseline and 5 specific clinical measures (ANO, ASIS, 5- and 17-domain NPC NSS) and concordant log_10_(CSF NfL) levels.

Longitudinal analyses were carried out via a linear mixed-effects model on the log-transformed serum NfL with miglustat therapy (yes vs no), IT HPβCD therapy (yes vs no), arimoclomol therapy (yes vs no), sex (male vs female), baseline age (years), the time elapsed since the baseline visit (years), and ANO (years), as fixed effect covariates, and patient ID as a random effect. Exponentiated estimated coefficients, 95% CIs, and *P* values describe the effects of the linear mixed-effects model. In the correlation analysis and the linear mixed-effects model, if multiple hypothesis tests were conducted (ie, testing correlation coefficients against 0 and regression model coefficients against 0), adjusted *P* values were obtained using the false discovery rate (FDR) correction.^35^ GraphPad Prism 10.1.1 was used to generate graphical figures.

### Modeling of serum NfL half-lives in individuals with NPC1

Baseline data for serum NfL were modeled for its biological decay in serum over 7 half-lives. The assumption made was that the rate of NfL production remains constant over time. Half-lives were calculated using the following equation:

$${NfL}_{\frac{1}{2}}\left(E,n\right)={NfL}_{Ctrl}+\left({NfL}_{0}-{NfL}_{Ctrl}\right)\times {\left(1-\left(\frac{0.5}{\frac{100}{E}}\right)\right)}^{n}$$

in which ${NfL}_{0}$ is the original NfL data point, ${NfL}_{Ctrl}$ is the average NfL value for control individuals (${NfL}_{Ctrl}$ = 0.6108), and ${NfL}_{\frac{1}{2}}\left(E,n\right)$ is the value of NfL after n half-lives and treatment with a drug that has efficacy E (all data points were log-transformed for the calculations). The above equation was applied to every original NfL data point using all combinations of n=1,2,3,4,5,6,7 and E=10%,20%,50%,100% to produce NfL half-life curves. For the statistical analysis of serum NfL half-lives, unpaired *t* tests were used to compare the distribution of NfL values at each combination of half-life and drug efficacy with the original NfL data set. For each drug efficacy, the first half-life to display a significant difference is listed, along with the *P* value from the *t* test.

## Results

### Study participant baseline clinical characteristics and demographics

Table 1 summarizes the baseline demographics and treatment status for both the control group (*n* = 24) and the NPC1 cohort (*n* = 118). Among these 118 individuals, 9 individuals had an undetermined ANO and were presumed presymptomatic at baseline. Fourteen individuals have an ASIS of 0 as assessed by a 0 score in the 17- and 5-domain NSS, indicative of a presymptomatic disease status.

Inclusion of some older adult NPC1 samples (range 0.3-68.1 years) resulted in a slightly higher, but not significant (*t* test, *P* = .213), mean age of the NPC1 cohort; 15.0 ± 14.8 years compared with 11.1 ± 4.8 years for the control group (range 2.1-19.4 years). Median ages were 10.6 years (IQR [3.8-20]) for the individuals with NPC1 and 11.0 years (IQR [7.4-15.1]) for the control group. In the NPC1 cohort, 62 (52.5%) were female compared with 8 (33.3%) in the control group (χ^2^, *P* = .117). The 9 individuals with NPC1 that had not yet developed neurological signs/symptoms had a mean age of 3.9 ± 5.7 years, (median [IQR] 1.1 [0.6-1.4] years, 5F, 4M).

At baseline, of the 118 NPC1 study participants, 55 (46.6%) were treated with miglustat, and 63 (53.4%) were not. Demographics and disease status of these 2 groups were comparable. The mean age between the 2 groups was comparable (*P* = .556), and the sex ratio was balanced in both groups. Mean ANO for those receiving miglustat was 7.5 ± 8.9 years (median [IRQ] 5.3 [2-9 years]) and those not receiving miglustat was 9.1 ± 12.0 years (median [IRQ] 5.0 [2-9] years) (*P* = .418, Table 1). There were also no significant differences between the miglustat treated and untreated groups for 17-domain NPC NSS (*P* = .845), 5-domain NPC NSS (*P* = .282), or ASIS (*P* = .349). At baseline, 3 individuals were treated with arimoclomol, and 8 were treated with IT HPβCD.

### Serum NfL levels in individuals with NPC1

**Figure 1:**
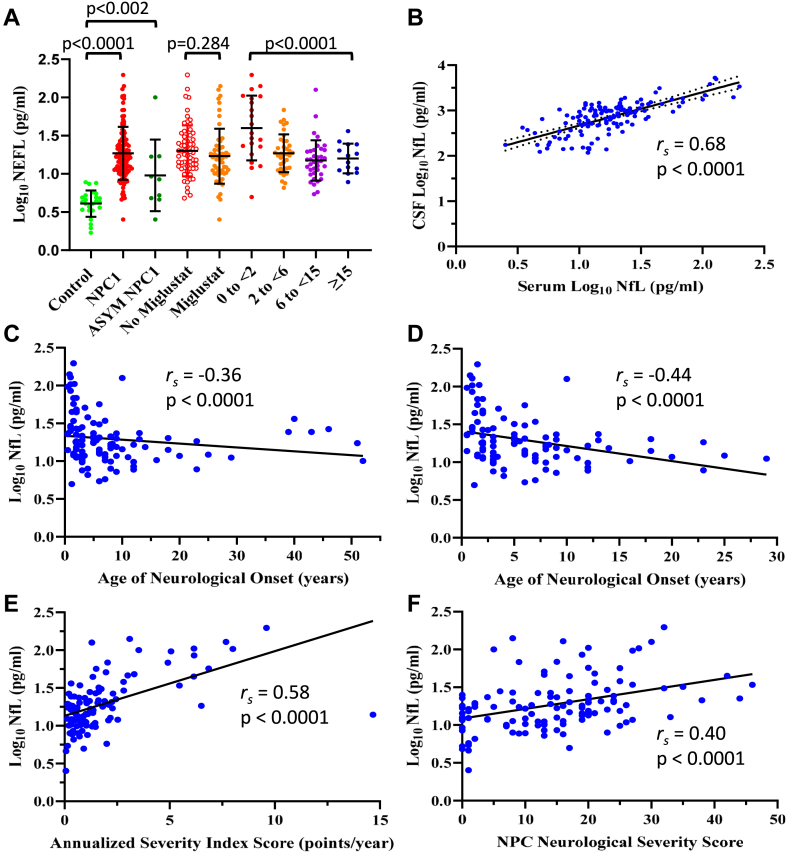
**Serum NfL levels in individuals with NPC1 and control individuals.** A. Baseline levels of NfL in individuals with NPC1 compared with age-appropriate control individuals. Included also are the baseline NfL levels from the 9 asymptomatic individuals. Additionally, analysis of individuals with NPC1 treated or not with miglustat and analysis of baseline data stratified into 4 age groups as defined by Vanier et al.^1^ B. Correlation graph of CSF versus serum NfL level. C and D. Correlation graph of serum NfL levels and age of neurological onset (ANO) in all individuals analyzed (C) and in individuals <30 years of age (D). E and F. Correlation graphs of serum NfL levels with ASIS (E) and NSS (F). ASIS, annual severity increment score; CSF, cerebrospinal fluid; NfL, neurofilament light; NPC1, Niemann-Pick disease, type C1; NSS, Neurological Severity Score.

The mean level for NfL in the NPC1 cohort (*n* = 118) was 27.0 ± 30.9 pg/mL (median [IRQ] 17.0 [11.4-23.3] pg/mL) and the mean level of NfL in the control group (*n* = 24) was 4.4 ± 1.6 pg/mL (median [IRQ] 4.3 [3.4-5.0] pg/m[), representing a 6.1-fold increase in mean levels of NfL in serum from individuals with NPC1 compared with pediatric controls. Log-transformation of the NfL values was performed to approximate normality of the data set and graphed (Figure 1A). Statistical analysis by unpaired *t* test showed the mean serum NfL level was elevated in the individuals with NPC1 at baseline compared with controls (*n* = 118, *P* < .0001). Raw NfL values (pg/mL) are presented as an additional file in Supplemental Table 1. The 9 asymptomatic individuals with NPC1 had a mean NfL level of 18.8 ± 29.3 pg/mL (median [IRQ] 8.5 [4.8-16.9] pg/mL) and was different from the control group (*n* = 24, *P* = .002, *t* test) and the symptomatic NPC1 group (*n* = 109, *P* = .0086, *t* test). Of these 9 individuals, 1 individual was identified as an outlier (interquartile test (Q3 + (1.5 × IRQ) >> Q3). Without this data point, the mean NfL level is 8.6 ± 5.2 pg/mL, *n* = 8 (median [IRQ] 6.9 [4.8-11.1] pg/mL). This represents a 1.9-fold higher mean NfL level in these 8 individuals compared with the controls (*t* test, *P* = .007). We also compared baseline levels of serum NfL in individuals with NPC1 being treated with miglustat (*n* = 55) or not (*n* = 63) (Figure 1A). No significant difference was observed (*t* test, *P* = .284). Baseline mean serum NfL levels in the treated cohort was 28.2 ± 31.7 pg/mL (median [IRQ] 16.5 [10.3-22.0] pg/mL) versus 25.6 ± 29.9 pg/mL (median [IRQ] 18.9 [11.9-29.4] pg/mL) for the untreated cohort.

The individuals with NPC1 were stratified into groups based on age of neurological onset as outlined previously.^1^ Mean serum NfL levels are lower in individuals with later neurological onset (Figure 1A, *P* < .0001, one-way analysis of variance). Values for the serum NfL within the age groups were as follows: 0 < 2 years (59.6 ± 50.9 pg/ml), 2 < 6 years (22.0 ± 22.0 pg/mL), 6 < 15 years (19.0 ± 20.2 pg/mL) and ≥ 15 years (17.5 ± 7.8 pg/mL).

In addition to NfL, the Neuro4-Plex assay used in this study also measured GFAP, TAU and UCHL-1 levels. In the case of GFAP, we found no difference between NPC1 and control samples (Supplemental Figure 1), similar to that reported previously.^21^ However, for both TAU and UCHL-1 there is a statistically significant increase, *P* = .025 and *P* = .046 respectively, in serum levels of these proteins in our cohort (Supplemental Figure 1). However, the increased level of these analytes is unlikely to be clinically relevant given the small increase and the wide overlapping range with the control group.

### Correlation of serum NfL levels with CSF NfL levels

To determine how well our serum NfL results correlated with CSF NfL levels, we compared 133 serum samples with their corresponding CSF samples obtained at the time of visit. We first analyzed the levels of NfL in CSF. Mean CSF NfL levels were significantly (*P* < .0001, Mann-Whitney U test) elevated in NPC1 samples (*n* = 133, 1018.04 ± 885.51 pg/mL) compared with age-appropriate comparison samples (172.21 ± 106 pg/mL). This represents a 5.9-fold increase of mean CSF NfL levels in NPC1 CSF (Supplemental Figure 2) and is consistent with a previous report utilizing a standard enzyme-linked immunoassay.^15^ These data were graphed against their corresponding serum values and the correlation between log(CSF NfL) and log(serum NfL) is presented in Figure 1B. The Spearman correlation coefficient is r_s_ = 0.68, *P* < .0001, demonstrating a strong correlation between CSF and serum levels of NfL.

### Correlation of serum NfL levels with clinical outcome measurements

The Spearman correlations between the log-transformed serum NfL levels at baseline and several clinical aspects of the disease are presented in Table 2 and Figure 1C-F. The results show moderate but highly significant correlations between log(serum NfL) levels and measures of both clinical severity (ANO and ASIS) and disease burden (5-and 17-domain NPC NSS), all with FDR-adjusted *P* ≤ .0001.

**Table 2:** Spearman correlation^a^ between log(Serum NfL) at baseline and prognostic measures of NPC1

Measurement	$r_{s}$ (95% CI)	FDR-Adjusted *P* Value	Raw *P* Value
Age of neuro onset (*n* = 109)	−0.36 (−0.51, −0.18)	.0001	.0001
ASIS (*n* = 104)	0.58 (0.43, 0.69)	<.0001	<.0001
5-domain severity score (*n* = 118)	0.43 (0.27, 0.56)	<.0001	<.0001
17-domain severity score (*n* = 118)	0.40 (0.24, 0.54)	<.0001	<.0001
log(CSF NfL) (*n* = 133)^b^	0.68 (0.58, 0.76)	<.0001	<.0001

*ASIS*, annual severity increment score; *CSF*, cerebrospinal fluid; *NfL*, neurofilament light.

a The Pearson correlation between log(serum NfL) and log(CSF NfL) was 0.715, based on 133 pairs of measurements. However, the normality checks revealed violations of normality assumption. Therefore, the Spearman correlation coefficient is presented here.

b Includes all serum samples and their available corresponding CSF samples.

A graphical representation and correlation analysis of the log(serum NfL) compared with ANO is presented in Figure 1C. The graph shows a negative correlation across the current cohort (r_s_ = −0.36, *P* < .0001). Of note, 6 samples were from individuals with ANO > 30 years, representing adult onset NPC1. When the results are replotted to exclude these older individuals, the correlation improves to (r_s_ = −0.44, *P* < .0001) (Fig. 1D). Analysis of log(serum NfL) compared with ASIS is presented in Figure 1E. This moderate to strong positive correlation is highly significant (r_s_ = 0.58, *P* < .0001). Figure 1F represents the data from the log(serum NfL) compared with 17-domain NSS. These results show a moderate correlation (r_s_ = 0.40, *P* < .0001) (Table 2).

### Effects of treatment on serum NfL levels in patients with NPC1

**Figure 2:**
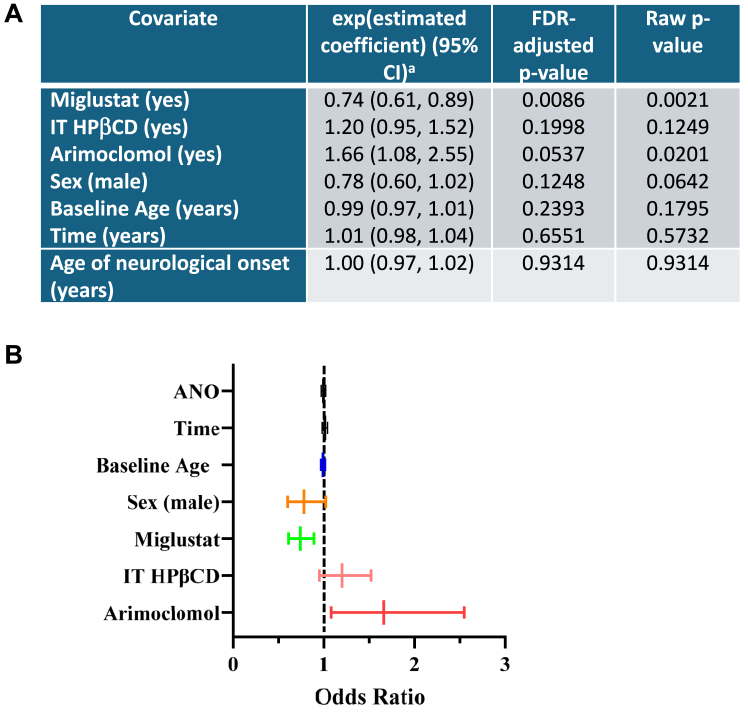
**Results of longitudinal analysis of log(serum NfL) using linear mixed-effects model (109 participants totaling 233 measurements).** A. Table representing a summary of the statistical analyses. The expression ^a^(exponentiated(estimated coefficient) − 1) × 100% reflects the percentage change in serum NfL levels for a 1-unit increase in the covariate. For example, an estimated coefficient of 0.74 for miglustat indicates that serum NfL levels decrease by 26% with miglustat treatment compared with without it. B. Forest plot of the effects of baseline age, time, ANO, sex, and treatment with miglustat, intrathecal HPβCD (adrabetadex, VTS270), and arimoclomol on serum NfL levels. ANO, age of neurological onset; HPβCD, hydroxypropyl-beta-cyclodextrin; NfL, neurofilament light.

A linear mixed-effect model was used to evaluate longitudinal serum NfL data. The results of this analysis are reported in Figure 2A and B. Fixed-effects covariates included sex, baseline age, the time elapsed since the baseline visit, ANO, and treatment status (miglustat, intrathecal adrabetadex (HPβCD) and arimoclomol). When all other variables are held constant, the introduction of miglustat treatment resulted in a reduction in the serum NfL levels by 26% on average, with a 95% CI of (11%, 39%) and FDR-adjusted *P* = .0086. The serum NfL level increases by 20% on average with the introduction of IT HPβCD, with a 95% CI of (−5% to 52%) (FDR-adjusted *P* = .1998).

Unexpectedly, the serum NfL level increases by 66% on average with the introduction of arimoclomol, with a 95% CI of (8%-155%) (FDR-adjusted *P* = .054). Because this may be due to the small sample size of 7 individuals treated with arimoclomol, we further analyzed longitudinal data to explore this result (Supplemental Figure 3). Considering when arimoclomol was initiated relative to the sample time points, serum NfL levels do not appear to be elevating in response to arimoclomol.

### Analysis of individuals with NPC1 before and after IT HPβCD treatment

**Figure 3:**
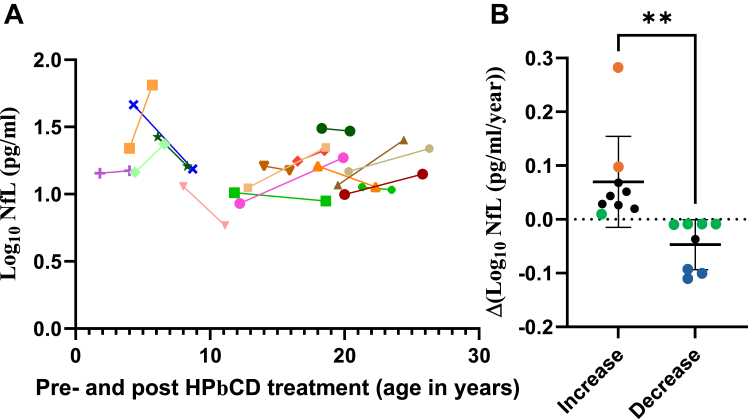
**Serum NfL levels in individuals with NPC1 treated with IT HPβCD.** A. Serum NfL levels in 17 individuals with NPC1 before and after IT HPβCD treatment. B. The 17 individuals were divided into those that had a calculated increase or decrease in NfL levels and plotted as the difference between before and after treatment normalized to the time interval between before and after treatment in years. Note that 5 of the 17 individuals showed small annual rates of change in serum NfL (green circles). Of the remainder, 3 of the 4 “responders” in the group showing a decrease in NfL received IT HPβCD treatment at <10 years of age (blue circles). In contrast, of the 8 remaining “nonresponders” showing an increase, 2 received IT HPβCD treatment at <10 years of age (orange circles). HPβCD, hydroxypropyl-beta-cyclodextrin; IT, intrathecal; NfL, neurofilament light; NPC1, Niemann-Pick disease, type C1.

We analyzed 17 individuals with NPC1 in the cohort in which a well-defined pre- and post-IT HPβCD treatment sample was available. Eight individuals showed reduced NfL levels, and 9 individuals showed increased NfL levels after initiation of IT HPβCD treatment (Figure 3A). Curiously, in the group of 6 individuals whose treatment started at <10 years of age, 3 individuals had reduced levels of NfL, 2 individuals had increased levels, and 1 appeared stable (Figure 3A). We reanalyzed these data for the amplitude of NfL change (increase or decrease) relative to the time interval in years from before to after treatment (Figure 3B). The results show a significant difference in response rate between the 2 groups (*P* < .0035). The mean age of the responder group was 12.5 ± 5.8 years (median [IRQ] 12.0 [7.5-18.1] years), whereas the nonresponder group was 11.9 ± 6.8 years (median [IRQ] 12.7 [4.4-19.5] years). Analysis of these subgroups comparing ANO, ASIS, and NSS did not provide further information because of the low numbers and variable scatter.

### Modeling of serum NfL half-lives from individuals with NPC1

The mean baseline levels of serum NfL in individuals with NPC1 was determined to be 27.0 ± 30.9 pg/mL. Using a model to predict biological half-life reduction of NfL under different treatment conditions (Supplemental Figure 4A), we modeled that the change in NfL levels from baseline based on drug efficacy to decrease the rate of NfL production. For a drug that is 100% or 50% effective, a statistically significant reduction in NfL levels could be detected after 1 half-life. For a drug that is 20% effective, 2 half-lives would be needed, and for drugs with 10% efficacy, it would take 4 half-lives before a meaningful difference in serum NfL could be detected compared with the baseline (Supplemental Figure 4B).

## Discussion

NPC1 lacks a robust serum biomarker that tracks well with neurological aspects of the disease. This study investigated serum neurofilament light (NfL) protein as a biomarker for NPC1 disease. Current data show that serum NfL levels are elevated in individuals with NPC1, and increased levels are associated with both increased disease severity and burden. Our data also show that serum NfL levels decrease with miglustat treatment, suggesting that it can be used to monitor treatment efficacy.

NfL is a component of the axonal cytoskeletal structure of neurons, and its presence in the CSF or serum of individuals indicates neuronal damage.^36^ Recently, NfL levels in blood were reduced in response to antisense oligonucleotide treatment of SOD1-dependent ALS, and the treatment was approved by the US FDA based in part on these NfL levels.^31^ Hence, the analysis of archived historical NPC1 serum samples for NfL from previous clinical trials could help ascertain its viability as a suitable serum biomarker for NPC1. Our results show that mean levels of NfL in serum of individuals with NPC1 (Table 1) are elevated by 6.1-fold compared with controls (Figure 1A), a value similar to the mean fold-difference found previously in the CSF of individuals with NPC1 and age-appropriate comparison groups (∼4-fold).^15^ This demonstrates a strong correlation between CSF NfL and serum NfL (Figure 1B), reflecting a confident understanding of the CNS neuropathology by analyzing serum NfL. The levels obtained here correspond very well with those reported previously for 26 individuals with NPC1 by Dardis et al^14^ and 11 individuals with NPC1 by Eratne et al^21^ and very recently for 49 individuals with NPC1 by Gonzalez-Ortiz et al.^22^

In our study, baseline NfL levels correlated with ANO, ASIS, and NPC NSS (Figure 1C-F, Table 2), indicating a close connection between NfL levels and NPC1 phenotypic parameters related to both disease severity (ANO and ASIS) and disease burden (NPC NSS). Of note, serum NfL levels were also significantly elevated in 9 presymptomatic individuals relative to control values. This is consistent with ongoing neurological damage before manifestation of neurological signs or symptoms and argues for early, even presymptomatic, intervention to slow NPC1 neuropathology.

Our results add to other evidence reported for swallowing^6^ and treatment outcomes and survival^4^^,^^5^ that demonstrate the benefit of miglustat in the treatment of NPC1. Our linear mixed-effects model analysis shows that miglustat has a significant effect on lowering NfL levels in serum by 26% (odds ratio = 0.74, 95% CI = 0.61-0.89, Figure 2A), consistent with a previous report showing a significant lowering of NfL in NPC1 CSF with miglustat treatment (odds ratio = 0.77, 95% CI = 0.62-0.96).^15^ Parenthetically, UCHL-1, another marker of nerve damage was also reduced in CSF of individuals with NPC1 who were on miglustat treatment (odds ratio = 0.70, 95% CI = 0.60-0.83),^12^ suggesting that UCHL-1 in serum may represent an addition marker of NPC1 neuropathology. In support of that, we found elevated serum levels of UCHL-1 in our multiplex assay (Supplemental Figure 1) and although the levels are small but significant, analysis of serum UCHL-1 combined with serum NfL may add extra statistical significance to monitoring NPC1 disease.

When we analyzed a group of individuals with NPC1 (*n* = 17) for pre- and post-IT HPβCD (Figure 3A), we observed approximately equal numbers of responders and nonresponders to IT HPβCD treatment (Figure 3B). Although 5 of 17 showed small annual rates of change in NfL after treatment, in the remaining individuals that showed decreased levels of NfL after treatment, 3 were <10 years of age, whereas in the group that showed an increase 2 were <10 years of age. Whether these results suggest that earlier treatment with IT HPβCD may provide greater benefit awaits further investigation. Conclusions related to efficacy of IT HPβCD are limited by the relatively small number of samples in this study. This limitation is also relevant to the assessment of arimoclomol.

NfL levels in sera of individuals with NPC1 serve as a marker of neuronal damage/death in the CNS. If a therapeutic intervention prevents such damage, we can estimate how long it would take to observe a significant reduction in NfL levels. Based on our model (Supplemental Figure 4), the time to detect that reduction will vary depending on the drug’s efficacy. If a drug is only 10% effective, it would take 4 half-lives to see a significant change in sera NfL levels. Given the reported half-life of serum NfL in humans ranges from 3 to 8 weeks^37^^,^^38^; therefore, assuming a maximum half-life of 2 months, we would expect to observe an effective NfL reduction after ∼8 months treatment with a drug that is 10% effective. This estimate assumes that the rate of generation of serum NfL is constant, but factors such as progression rate of the disease [d(NSS)/dt] could affect these calculations.

Our data presented here are congruent with the notion that serum NfL can be used as a biomarker for NPC1 neurological disease progression and therapeutic intervention. If approved by the FDA as such, it will provide hope for the NPC1 community and a clear direction forward to identify new therapeutics through clinical trials.

## Data Availability

The data presented in this study are present in this manuscript and are available to researchers with ethical approval upon request consistent with National Institutes of Health intramural research policies.

## Conflict of Interest

The authors declare no conflicts of interest.
