# Supplementary material for: Serum neurofilament light protein as a biomarker in Niemann-Pick disease, type C1
Source: Genet Med Open. 2025 Jul 7;3:103443. doi: 10.1016/j.gimo.2025.103443 (PMC12351337; doi:10.1016/j.gimo.2025.103443)
Supplement: Supplemental Figures [file mmc1.pptx]

## Slide 1
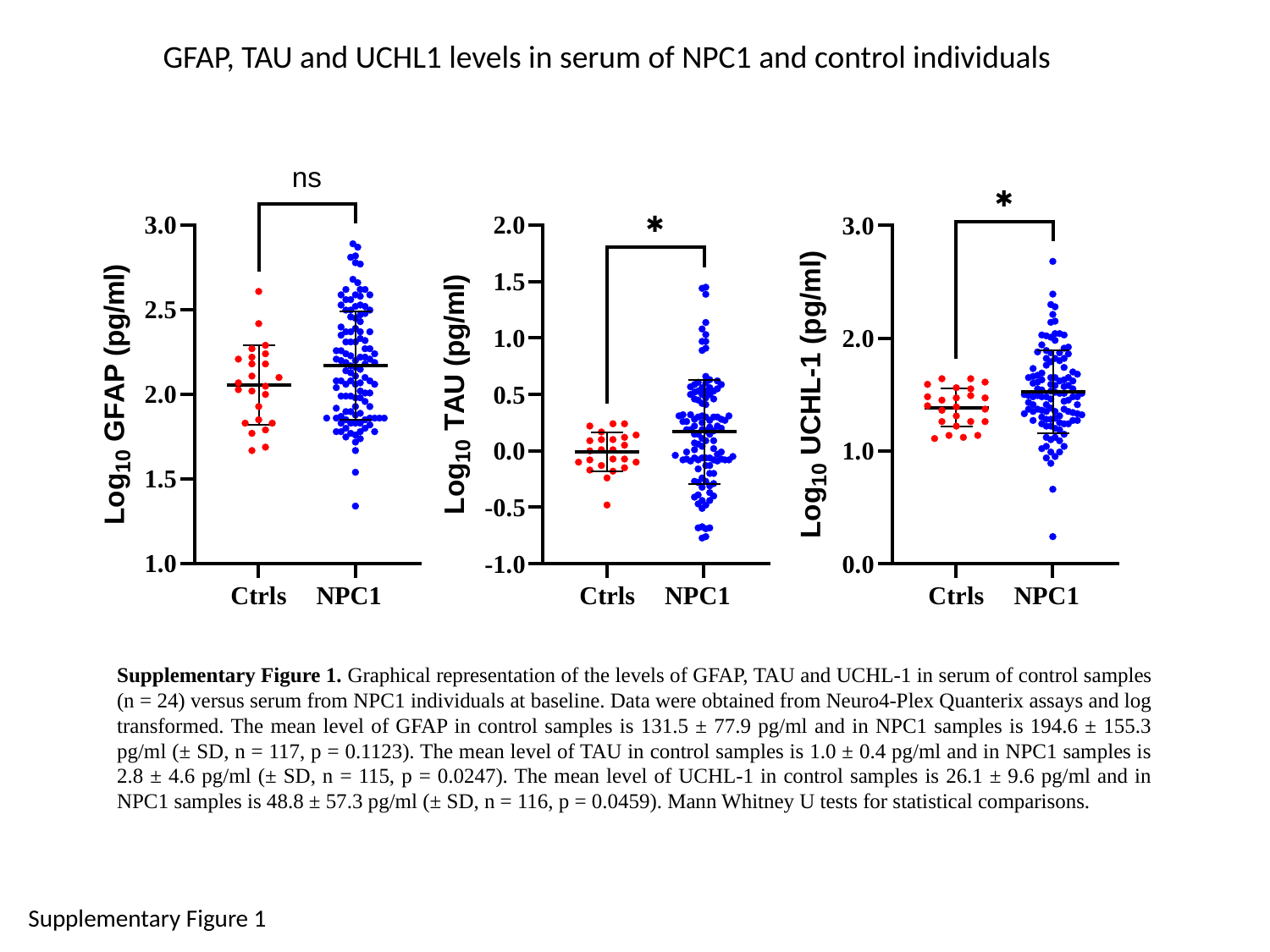

Log10 of GFAP, TAU and UCHL-1 Levels in Control and NPC1 Serum Samples
GFAP
TAU
UCHL-1
GFAP, TAU and UCHL1 levels in serum of NPC1 and control individuals
Supplementary Figure 1. Graphical representation of the levels of GFAP, TAU and UCHL-1 in serum of control samples (n = 24) versus serum from NPC1 individuals at baseline. Data were obtained from Neuro4-Plex Quanterix assays and log transformed. The mean level of GFAP in control samples is 131.5 ± 77.9 pg/ml and in NPC1 samples is 194.6 ± 155.3 pg/ml (± SD, n = 117, p = 0.1123). The mean level of TAU in control samples is 1.0 ± 0.4 pg/ml and in NPC1 samples is 2.8 ± 4.6 pg/ml (± SD, n = 115, p = 0.0247). The mean level of UCHL-1 in control samples is 26.1 ± 9.6 pg/ml and in NPC1 samples is 48.8 ± 57.3 pg/ml (± SD, n = 116, p = 0.0459). Mann Whitney U tests for statistical comparisons.
Supplementary Figure 1
Cawley et al. Supplementary Figure 2

## Slide 2
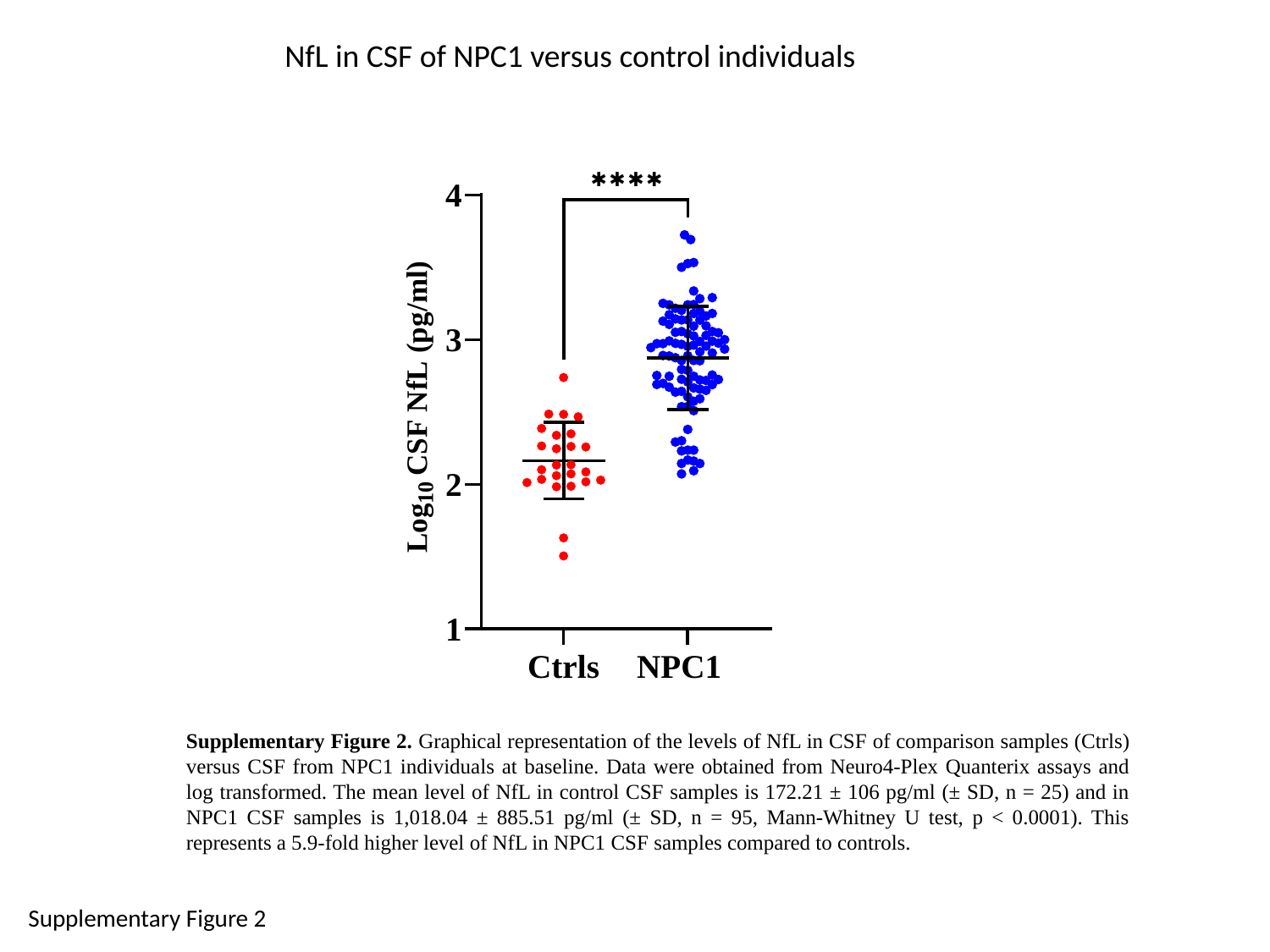

Log10 (CSF NfL) Levels in Control and NPC1 CSF samples
NfL in CSF of NPC1 versus control individuals
Supplementary Figure 2. Graphical representation of the levels of NfL in CSF of comparison samples (Ctrls) versus CSF from NPC1 individuals at baseline. Data were obtained from Neuro4-Plex Quanterix assays and log transformed. The mean level of NfL in control CSF samples is 172.21 ± 106 pg/ml (± SD, n = 25) and in NPC1 CSF samples is 1,018.04 ± 885.51 pg/ml (± SD, n = 95, Mann-Whitney U test, p < 0.0001). This represents a 5.9-fold higher level of NfL in NPC1 CSF samples compared to controls.
Supplementary Figure 2
Cawley et al. Supplementary Figure 1

## Slide 3
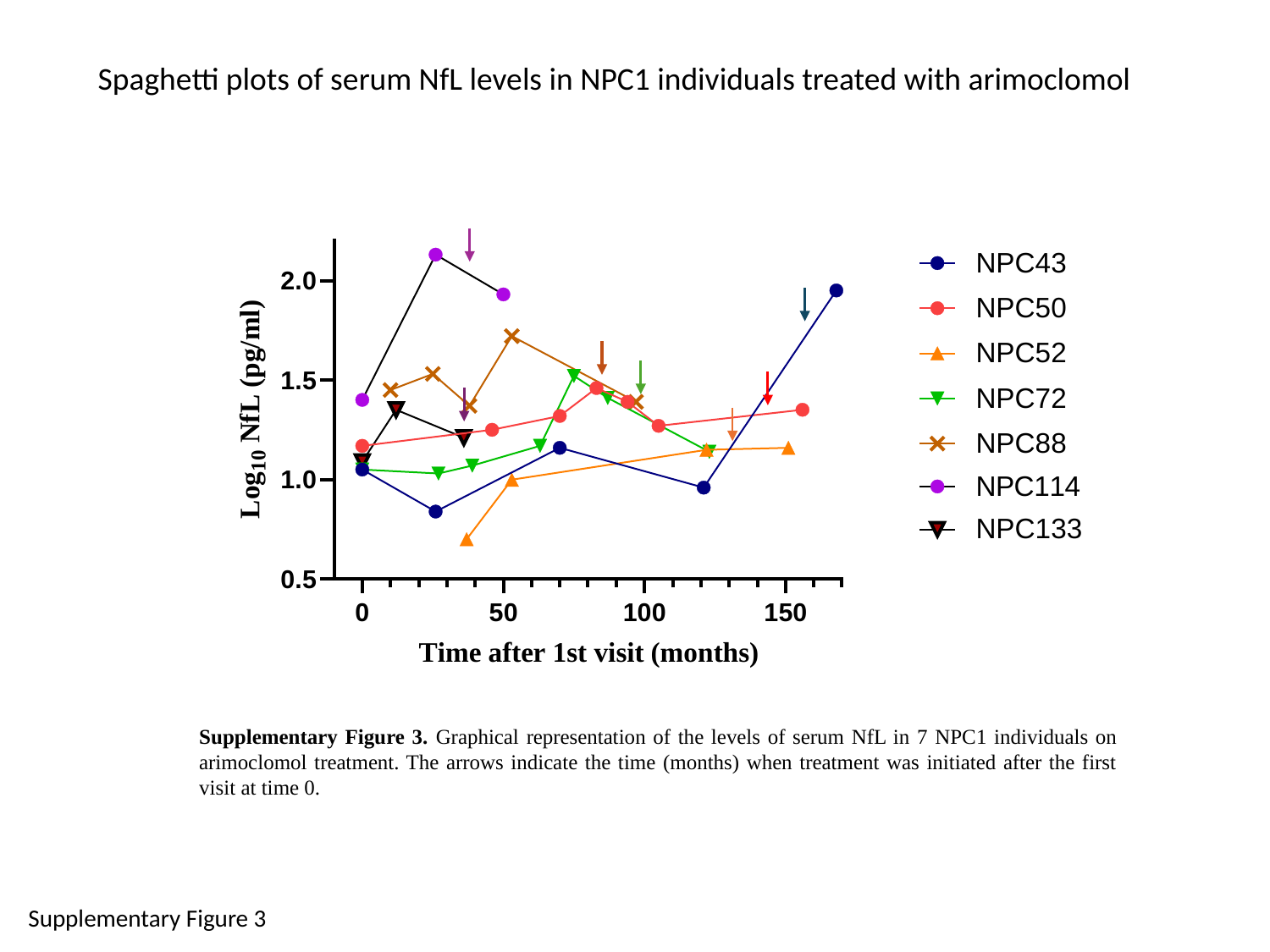

Spaghetti plots of serum NfL levels in NPC1 individuals treated with arimoclomol
Supplementary Figure 3. Graphical representation of the levels of serum NfL in 7 NPC1 individuals on arimoclomol treatment. The arrows indicate the time (months) when treatment was initiated after the first visit at time 0.
Supplementary Figure 3
Cawley et al. Supplementary Figure 3

## Slide 4
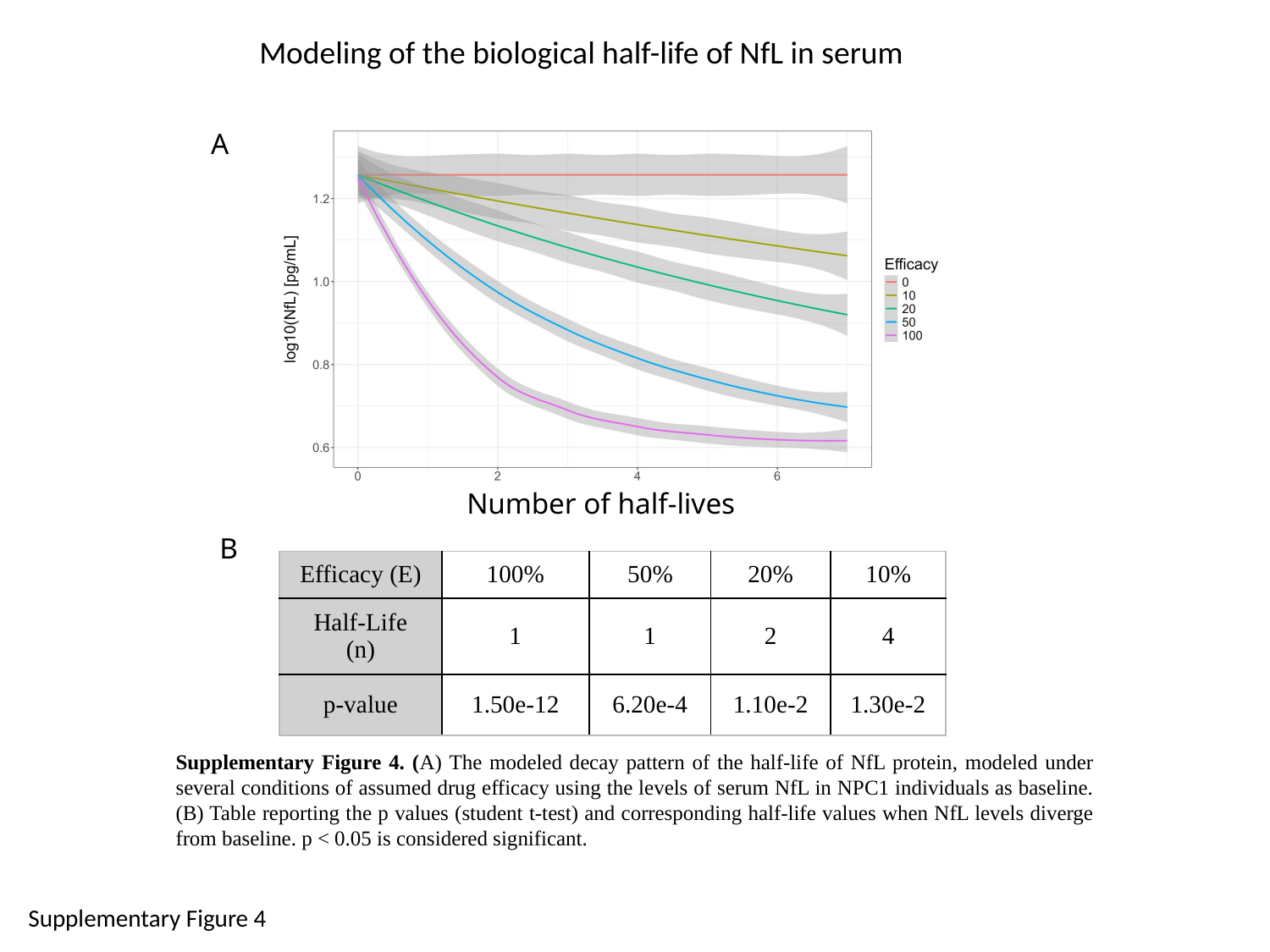

Modeling of the biological half-life of NfL in serum
A
Number of half-lives
B
| Efficacy (E) | 100% | 50% | 20% | 10% |
| --- | --- | --- | --- | --- |
| Half-Life (n) | 1 | 1 | 2 | 4 |
| p-value | 1.50e-12 | 6.20e-4 | 1.10e-2 | 1.30e-2 |
Supplementary Figure 4. (A) The modeled decay pattern of the half-life of NfL protein, modeled under several conditions of assumed drug efficacy using the levels of serum NfL in NPC1 individuals as baseline. (B) Table reporting the p values (student t-test) and corresponding half-life values when NfL levels diverge from baseline. p < 0.05 is considered significant.
Supplementary Figure 4
